# Contribution of Glutamatergic and GABAergic Mechanisms to the Plasticity‐Modulating Effects of Dopamine in the Human Motor Cortex

**DOI:** 10.1002/hbm.70162

**Published:** 2025-02-13

**Authors:** Elham Ghanavati, Mohammad Ali Salehinejad, Marie C. Beaupain, Lorena Melo, Amba Frese, Min‐Fang Kuo, Michael A. Nitsche

**Affiliations:** ^1^ Department of Psychology and Neurosciences Leibniz Research Centre for Working Environment and Human Factors (IfADo) Dortmund Germany; ^2^ Department of Psychology Ruhr University Bochum Bochum Germany; ^3^ School of Cognitive Sciences Institute for Research in Fundamental Sciences Tehran Iran; ^4^ International Graduate School of Neuroscience, Ruhr University Bochum Bochum Germany; ^5^ Bielefeld University, University Hospital OWL, Protestant Hospital of Bethel Foundation University Clinic of Psychiatry Psychotherapy Bielefeld Germany; ^6^ German Center for Mental Health (DZPG) Bochum Germany

**Keywords:** cortical excitability, dopamine, neuroplasticity, transcranial direct current stimulation, transcranial magnetic stimulation

## Abstract

Dopamine, a key neuromodulator in the central nervous system, regulates cortical excitability and plasticity by interacting with glutamate and GABA receptors, which are affected by dopamine receptor subtypes (D1‐ and D2‐like). Non‐invasive brain stimulation techniques can induce plasticity and monitor cortical facilitation and inhibition in humans. In a randomized, placebo‐controlled, double‐blinded study, we investigated how dopamine and D1‐ and D2‐like receptors impact transcranial direct current stimulation (tDCS)‐induced plasticity concerning glutamatergic and GABAergic mechanisms. Eighteen healthy volunteers received 1 mA anodal (13 min) and cathodal tDCS (9 min) over the left motor cortex combined with the dopaminergic agents l‐dopa (general dopamine activation), bromocriptine (D2‐like receptor agonist), combined D2 antagonism via sulpiride and general dopaminergic activation via l‐dopa to activate D1‐like receptors, and placebo medication. Glutamate‐related cortical facilitation and GABA‐related cortical inhibition were monitored using transcranial magnetic stimulation techniques, including I–O curve, intracortical facilitation (ICF), short‐interval intracortical inhibition (SICI), and I‐wave facilitation protocols. Our results indicate that anodal tDCS alone enhanced the I–O curve and ICF while decreasing SICI. Conversely, cathodal tDCS decreased the I‐O curve and ICF while increasing SICI. General dopamine and D2 receptor activation combined with anodal tDCS decreased the I‐O curve and ICF, but enhanced SICI compared to tDCS alone. When paired with cathodal tDCS, general dopamine and D2‐like receptor activity enhancement prolonged the cathodal tDCS effect on excitability. After anodal tDCS, D1‐like receptor activation increased the I‐O curve and ICF while reducing SICI. These effects were abolished with cathodal tDCS. Dopaminergic substances combined with anodal and cathodal tDCS did not have a significant effect on I‐wave facilitation. These results suggest that D1‐like receptor activation enhanced LTP‐like plasticity and abolished LTD‐like plasticity via glutamatergic NMDA receptor enhancement, while global dopaminergic and D2‐like receptor enhancement weakened LTP‐like but strengthened LTD‐like plasticity primarily via glutamatergic NMDA receptor activity diminution.

## Graphical abstract

Background: Dopamine modulates cortical excitability and plasticity by influencing glutamate and GABA receptor activity. This study investigates the impact of general dopaminergic activation and D1‐ and D2‐like receptor modulation on transcranial direct current stimulation (tDCS)‐induced plasticity in humans.Methods:• Pharmacological intervention: l‐dopa (general DA activation), Bromocriptine (D2 agonist), Sulpiride + l‐dopa (D1‐like activation), and placebo.• tDCS: Anodal (1 mA, 13 min) and Cathodal (1 mA, 9 min) over the left motor cortex.• Neurophysiological measures: Glutamatergic activity (I‐O curve, ICF) and GABAergic activity (SICI, I‐wave facilitation).Results:• D2‐like receptor and global dopamine activation + anodal tDCS: decreased the I‐O curve and ICF, but enhanced SICI• D2‐like receptor and global dopamine activation + cathodal tDCS: prolonged the cathodal tDCS effect on SICI‐ICF and IO‐curve• D1‐like receptor activation + anodal tDCS: increased the I‐O curve and ICF while reducing SICI• D1‐like receptor activation + cathodal tDCS: abolished cathodal tDCS effects• No significant effect of dopaminergic substances combined with anodal and on I‐wave facilitationConclusion:D1‐like receptor activation enhanced LTP‐like plasticity and abolished LTD‐like plasticity via glutamatergic NMDA receptor enhancement, while global dopaminergic and D2‐like receptor enhancement weakened LTP‐like but strengthened LTD‐like plasticity primarily via glutamatergic NMDA receptor activity diminution.

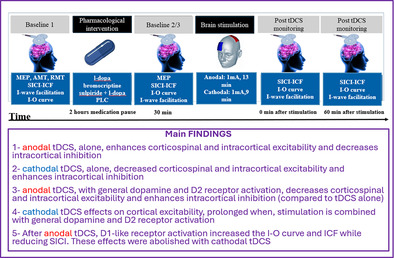

## 1 Introduction

Dopamine (DA), a crucial neuromodulator in the brain, plays a pivotal role in various aspects of neural plasticity and contributes to a multitude of neural and cognitive functions, including attention, learning, motivation, and memory formation (Robbins 2003; Speranza et al. 2021; Wise 2004). Dysfunctions of the dopaminergic system have been linked to the pathophysiology of various neurological and psychiatric diseases, such as schizophrenia, Parkinson's disease, and attention‐deficit/hyperactivity disorder (Calabresi et al. 2015; Grace 2012; Wu et al. 2012). A key mechanism of these DA effects involves the modulation of synaptic plasticity (Asl et al. 2019; Calabresi et al. 2007). Plasticity can be categorized into long‐term potentiation (LTP) through synaptic strengthening and long‐term depression (LTD) arising from the weakening of synaptic connections, both crucial elements in learning and memory formation (Citri and Malenka 2008; Whitlock et al. 2006). DA significantly contributes to the establishment of both LTP and LTD (Pedrosa and Clopath 2017).

Dopaminergic modulation of synaptic plasticity is complex, non‐linear, depends on D1‐ and D2‐like receptor subtypes, and affects both excitatory glutamatergic and inhibitory GABAergic mechanisms (Ghanavati et al. 2022; Marino and Levy 2019; Seamans and Yang 2004). Glutamatergic *N*‐methyl‐d‐aspartate (NMDA) receptors are critical for synaptic plasticity and determine the direction of plasticity by controlling intracellular calcium concentration alterations (Citri and Malenka 2008; Lisman 2001). Numerous studies have been conducted to understand the bidirectional regulation of NMDA receptors accomplished by DA (Cepeda and Levine 2006; Seamans and Yang 2004). The interaction between DA and GABA receptors is also crucial for cortical inhibition, which, beyond the glutamatergic system, is involved in the regulation of executive and cognitive functions and the induction of plasticity (Seamans et al. 2001; Trantham‐Davidson et al. 2004).

It is suggested that D1 activity strengthens NMDA and GABA activity, yet for LTP‐inducing cerebral activity states, NMDA receptor activity predominates (Seamans et al. 2001). In accordance, glutamate‐dependent LTP is strengthened by D1‐dependent potentiation of NMDA receptor activity (Li et al. 2010; Varela et al. 2009). On the other hand, D2 receptor activity decreases NMDA and GABA activity, and low NMDA receptor activation promotes LTD (Higley and Sabatini 2010; Li et al. 2009; Seamans and Yang 2004).

Non‐invasive brain stimulation (NIBS) techniques are increasingly used to investigate cortical excitability and induce neuroplasticity in the human brain (Polanía et al. 2018). Transcranial direct current stimulation (tDCS) is a safe (Salehinejad and Siniatchkin 2024) neuromodulatory technique that alters neuronal excitability via low‐intensity electrical currents applied to the brain, with prolonged stimulation inducing LTP‐ and LTD‐like plasticity (Nitsche et al. 2008; Nitsche and Paulus 2011; Stagg and Nitsche 2011). tDCS‐induced plasticity depends on GABAergic and glutamatergic mechanisms (Heimrath et al. 2020; Nitsche, Fricke, et al. 2003; Stagg et al. 2009). tDCS‐generated LTP‐ and LTD‐like after‐effects are abolished by dextromethorphan (DMO), an NMDA receptor antagonist (Nitsche, Fricke, et al. 2003), and LTP‐like plasticity is prolonged via the partial NMDA receptor agonist D‐cycloserine (CYC) (Ghanavati et al. 2022; Nitsche, Jaussi, et al. 2004). Furthermore, GABA reduction might have a gating function for tDCS‐induced plasticity since GABA is reduced by both anodal and cathodal tDCS (Stagg et al. 2009). A modulatory effect of DA on tDCS‐induced neuroplasticity has been identified. General DA and D2‐like receptor activity enhancement reversed anodal tDCS‐induced LTP‐like plasticity into LTD‐like plasticity, while it prolonged cathodal tDCS‐induced LTD‐like plasticity (Fresnoza, Stiksrud, et al. 2014; Kuo et al. 2007). In a recent study, we showed that the LTP‐like plasticity reduction accomplished by general DA activation is probably caused by a D2‐like receptor‐dependent reduction of NMDA receptor activity since NMDA receptor enhancement by CYC restored respective LTP‐like plasticity (Ghanavati et al. 2022). In contrast, the excitability increase induced by anodal tDCS was preserved under D1‐like receptor activation, while the excitability‐diminishing effect of cathodal tDCS was abolished (Fresnoza, Paulus, et al. 2014), in principle accordance with an NMDA receptor activity enhancement accomplished by D1‐like receptor activation. Nevertheless, the underlying mechanisms are incompletely understood and the precise contributions of D1‐ and D2‐like receptors to tDCS‐induced plasticity remain unclear. Clarifying these effects is vital for understanding dopaminergic modulation of cortical excitability, including plasticity, to enhance mechanistic understanding and to improve on this foundation neuromodulatory treatments for DA‐related and other disorders of the brain by adjusting stimulation protocols to pathologic alterations of the dopaminergic system, dopaminergic/anti‐dopaminergic medication, as well as to develop synergistic interventions involving brain stimulation and pharmacological interventions tackling the dopaminergic system.

Transcranial magnetic stimulation (TMS) can be applied with different paradigms to provide information about neurotransmitter systems involved in cortical excitability and plasticity (e.g., glutamatergic, GABAergic, and cholinergic systems) (Pascual‐Leone et al. 1998; Salehinejad et al. 2021). Intracortical facilitation (ICF) is regulated by glutamate‐mediated cortical facilitation as it is reduced by the NMDA blocker dextromethorphan (Chen 2000; Ziemann, Chen, et al. 1998). Short‐interval intra‐cortical inhibition (SICI) is primarily GABA‐mediated with some glutamate involvement, enhanced by GABA receptor agonists and NMDA receptor antagonists (Ziemann, Chen, et al. 1998). I‐wave facilitation is regulated by GABA‐related inhibition, shown by decreased I‐wave facilitation under GABA agonists (Ziemann, Tergau, et al. 1998). Another TMS protocol, the input–output (I–O) curve, which monitors corticospinal neuron excitability, is influenced by glutamatergic and adrenergic transmission at higher TMS intensities (for a comprehensive review, see Chen 2000 and Paulus et al. 2008).

Since tDCS‐induced neuroplasticity is suggested to depend on both glutamatergic and GABAergic mechanisms (Heimrath et al. 2020; Nitsche, Fricke, et al. 2003; Stagg and Nitsche 2011), we assumed that DA affects it via these mechanisms and that the respective effects depend on the specific receptor subtype (D1‐like, D2‐like) (Seamans and Yang 2004). Accordingly, in the present study, we aimed to explore the contribution of glutamatergic and GABAergic mechanisms to the plasticity‐modulating effects of DA and D1‐ and D2‐like DA receptor subtypes in the human motor cortex. To this end, we applied the DA precursor l‐dopa, the D2 receptor agonist bromocriptine, and a combination of the D2‐like receptor antagonist sulpiride and l‐dopa for activating D1‐like receptors combined with anodal and cathodal tDCS to induce LTP‐ and LTD‐like plasticity. Based on previous research, we hypothesized that for LTP‐like plasticity, in the case of global DA and D2 receptor activation, the NMDA receptor activity‐reducing effect controlled by D2 receptors dominates, leading to decreased I/O curves and ICF, and increased SICI, while GABA reduction might result in I‐wave enhancement. Conversely, D1‐like receptors enhance NMDA and GABA receptor activity, but the NMDA receptor‐enhancing effect predominates in LTP induction scenarios, resulting in increased I/O curves and ICF, reduced SICI, while I‐wave facilitation might be reduced because of an additional enhancement of GABA activity. For LTD‐like plasticity, we hypothesized that DA and D2‐like receptors stabilize it by reducing NMDA receptor activity, leading to decreased ICF and I/O curves and enhanced SICI, while GABA reduction enhances I‐wave facilitation. Conversely, D1‐like receptors may counteract LTD‐like plasticity by enhancing NMDA receptor activity, causing increased ICF, decreased SICI, increased I/O curves, but decreased I‐wave facilitation through GABA enhancement.

## 2 Methods

### 2.1 Participants

We recruited 18 healthy, right‐handed, non‐smoking individuals (10 females, mean age: 28.72 ± 5.51 years). The sample size was determined using a power analysis for the primary outcome parameter, the interactions of repeated measure ANOVAs with the within‐subject factors plasticity direction, medication, and time point, and TMS measures as the dependent variable. This analysis estimated that a minimum of 10 participants would be required for a critical *α* error level of 0.05, a *β* error of 0.05, and a small to medium effect size of *f* = 0.15. To account for potential dropouts and variability, eight additional participants were included, resulting in a total sample size of 18. This sample size aligns with those of prior studies combining central nervous system‐active substances with tDCS or paired associative stimulation to investigate neuroplastic mechanisms, which resulted in significant effects and meaningful differences between experimental conditions (Fresnoza, Stiksrud, et al. 2014; Korchounov et al. 2007; Melo et al. 2024; Silverstein et al. 2019). All participants underwent a medical examination to assess their health state, contraindications for NIBS, and medication intake. The study was conducted following the Declaration of Helsinki and was approved by the Institutional Review Board of the Leibniz Research Centre for Working Environment and Human Factors, Dortmund, Germany. All participants gave written informed consent before inclusion and were financially compensated.

### 2.2 Cortical Excitability Monitoring With TMS

Single and paired‐pulse TMS protocols were used to assess corticospinal and intracortical motor cortex excitability, including single‐pulse motor‐evoked potentials (MEP), resting motor threshold (RMT), active motor threshold (AMT), I–O curve, SICI–ICF, and intracortical I‐wave facilitation, following prior research (Salehinejad, Ghanavati, Reinders, et al. 2022; Salehinejad et al. 2021). MEP, RMT, AMT, and the I–O curve examine corticospinal excitability; SICI–ICF and I‐wave facilitation measure intracortical excitability. Single‐pulse monophasic TMS at 0.25 Hz ± 10% (random jitter) was delivered by a Magstim 200 stimulator (Magstim Company Ltd., Spring‐Gardens, Whitland, UK) through a figure‐of‐eight magnetic coil (outer diameter of one winding 70 mm; peak magnetic field 2 T). For the paired‐pulse protocols, the coil was connected to two Magstim 200 stimulators by a bistim module. The coil was held at a 45° angle to the midline over the left primary motor cortex. Surface MEPs were recorded from the right abductor digiti minimi muscle (ADM) with gold cup electrodes in a belly‐tendon montage. Signals were amplified and filtered (1000; 3 Hz–3 kHz) using a D440‐2 amplifier (Digitimer, Welwyn Garden City, UK) and were digitized (sampling rate 5 kHz) with a micro 1401 AD converter (Cambridge Electronic Design, Cambridge, UK), controlled by Signal Software (Cambridge Electronic Design, v. 2.13). RMT was obtained by the TMS Motor Threshold Assessment Tool (MTAT 2.0, http://www.clinicalresearcher.org/software.%20html) and was determined as the lowest stimulator intensity required to evoke a peak‐to‐peak MEP of 50 μV in the relaxed ADM in at least five out of 10 consecutive trials. AMT was determined as the lowest stimulator intensity required to elicit an MEP response of ∼200–300 μV during moderate tonic contraction of the right ADM (∼20% of maximum muscle strength) in at least three of six consecutive trials.

#### 2.2.1 I–O Curve

The I–O curve, a single‐pulse TMS protocol, reflects corticospinal excitability through increasing MEP amplitudes with higher TMS intensity (Chen 2000) which are linked to glutamatergic transmission (Paulus et al. 2008). MEPs were measured in the relaxed right ADM muscle across four intensity blocks (100%, 110%, 130%, 150% RMT) (Batsikadze et al. 2013), with 15 pulses per block, and mean MEPs were calculated for each intensity.

#### 2.2.2 Intracortical Inhibition and Facilitation (SICI and ICF)

SICI and ICF, acquired through paired‐pulse TMS, assess intracortical excitability. SICI mainly evaluates GABA‐controlled inhibition with some glutamate impact (Stagg et al. 2011; Ziemann et al. 1996a), while ICF monitors glutamatergic ICF (Stagg et al. 2011; Ziemann, Chen, et al. 1998). A conditioning stimulus (intensity set at 70% of AMT) was followed by a test stimulus adjusted to produce an MEP of ∼1 mV amplitude (set at SI1 mV). If required, TMS intensity for the test stimulus was readjusted to compensate for substance or tDCS effects on corticospinal excitability (Batsikadze et al. 2013; Nitsche et al. 2005). Paired pulses were applied at ISIs of 2, 3, 5, 10, and 15 ms, with 2, 3, and 5 ms producing inhibitory effects, and 10 and 15 ms producing facilitatory effects on test stimulus amplitude (Kujirai et al. 1993). The paired pulses and one single test pulse were delivered in pseudorandomized ordered blocks every 4 ± 0.4 s, in which each ISI and the single test pulse were applied once. These blocks were repeated 15 times, resulting in 90 stimuli.

#### 2.2.3 I‐Wave Facilitation

This TMS protocol monitors I (indirect) waves, which refer to high‐frequency repetitive discharges of corticospinal neurons triggered by motor cortex stimulation (Di Lazzaro et al. 2012; Ziemann, Tergau, Wassermann, et al. 1998). In this protocol, two successive stimuli are delivered. The intensity of the first conditioning stimulus is adjusted to produce a baseline MEP of ∼1 mV, followed by a second stimulus set to 70% RMT (Ziemann, Tergau, Wassermann, et al. 1998). Paired pulses were applied at ISIs of 1.1, 1.3, 1.5, 2.3, 2.5, 2.7, and 2.9 ms, with facilitatory effects at ISIs with peaks at about 1.3 and 2.6 ms (Ziemann, Tergau, Wassermann, et al. 1998). This effect is suggested to originate from I‐waves elicited by the subthreshold stimulus and is controlled by GABA‐related neural circuits (Paulus et al. 2008; Ziemann, Tergau, Wassermann, et al. 1998). The pairs of stimuli were pseudorandomized and organized in blocks in which each double‐pulse stimulus with a specific ISI and one single test stimulus was applied once every 4 ± 0.4 s. These blocks were repeated 15 times, resulting in 120 stimuli.

### 2.3 Pharmacological Intervention

To explore the effect of DAergic substances on cortical excitability and plasticity, We administered 100 mg of l‐dopa (*Levodopa/Benserazide* 100/25 mg, *neuraxpharm* Pharma, Langenfeld, Germany) for global DA activation, 10 mg of bromocriptine (*Pravidel* 2.5 mg, *Viatris* Pharma, Troisdorf, Germany) for D2‐like receptor activation, 400 mg of the D2 antagonist sulpiride (*Sulpirid 200 mg, neuraxpharm* Pharma, Langenfeld, Germany) combined with 100 mg l‐dopa for D1‐like receptor activation, or equivalent placebo (PLC) medication (*P‐Tabletten weiß* 8 mm *Lichtenstein*, *Zentiva* Pharma, Frankfurt, Germany). All substances were administered orally. We placed all medications, including the placebo and active drugs, inside identical capsules to ensure consistency, blinding, and resemblance in shape and administration. These dosages of the respective active substances resulted in plasticity induction procedure‐dependent effects in previous studies (Fresnoza, Paulus, et al. 2014; Fresnoza, Stiksrud, et al. 2014; Kuo et al. 2007; Nitsche et al. 2009).

### 2.4 tDCS

Low‐intensity direct current was employed by a battery‐driven constant current stimulator (NeuroConn, Germany) through a pair of conductive rubber electrodes embedded in saline‐soaked sponges (35 cm^2^). The target electrode was placed over the motor cortex hotspot of the right ADM (identified by TMS) at an angle of 45° from the midsagittal line, and the return electrode was positioned over the contralateral supraorbital region. Stimulation was applied with 1 mA for 13 min (anodal) or 9 min (cathodal) tDCS, which resulted in after‐effects of about 1‐h duration in previous studies (Nitsche, Liebetanz, et al. 2003; Nitsche and Paulus 2001). A 15 s ramping‐up and ‐down of stimulation intensity at the start and end of the intervention was applied to reduce stimulation perception (Nitsche et al. 2008).

### 2.5 Experimental Procedure

**FIGURE 1:**
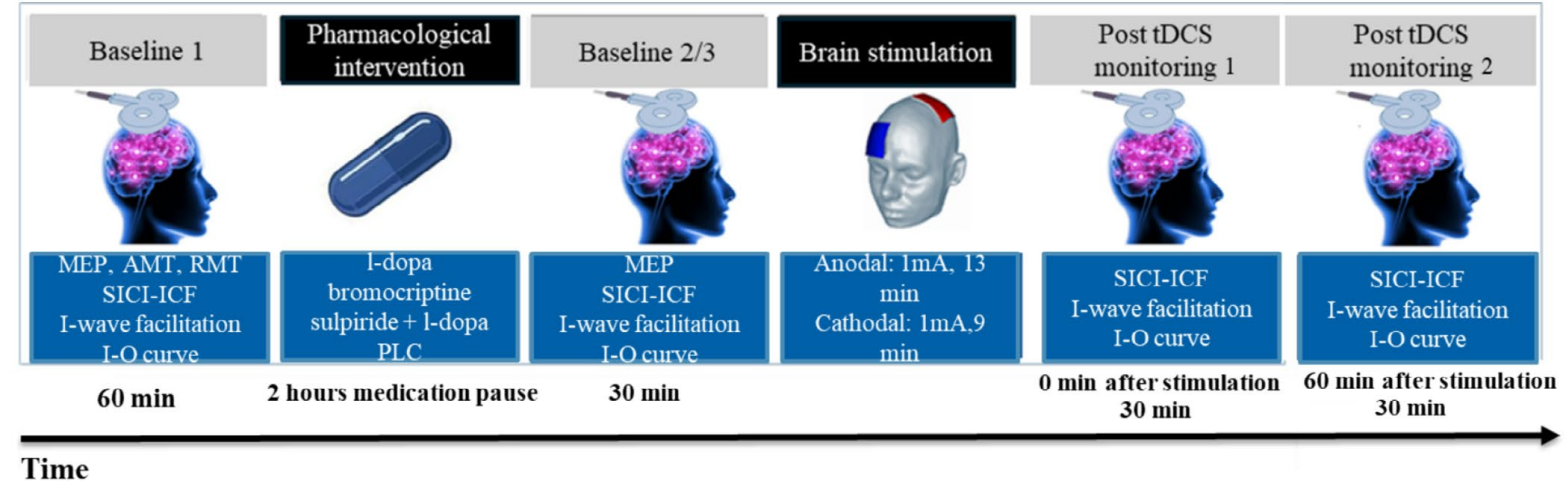
Schematic representation of the experimental procedure. In each session, participants received a combination of tDCS and medication. SICI and ICF, I‐wave facilitation, and the I–O curve were monitored before tDCS, and before and after medication (baseline 1 and 2/3) via TMS‐induced motor evoked potentials. tDCS after‐effects were monitored via the same recordings immediately and 1 h after stimulation.

This study used a crossover, randomized, placebo‐controlled, partially double‐blinded design. Participants were blinded to medication and tDCS conditions, and the experimenter was blinded to the medication condition. Participants attended eight sessions, each at least 1 week apart to minimize carry‐over effects. During each session, participants received a specific pharmacological intervention (placebo, l‐dopa, bromocriptine, or sulpiride with l‐dopa) combined with anodal or cathodal tDCS in randomized order, ensuring all combinations were tested in each participant. Each session followed the same steps, as shown in Figure 1. Sessions were scheduled flexibly, typically starting between 10:00 a.m. and 12:00 p.m., with drug administration occurring approximately an hour later, to ensure flexibility and consistent timing. They sat in a comfortable reclining chair with head‐ and armrests, with an inflatable pillow around their necks for head stabilization. First, the motor cortex hotspot was determined (the coil position over the primary motor area that produces the largest MEP in the right ADM with a given medium TMS intensity), and a waterproof pen marked the position of the TMS coil and ADM electrodes to guarantee stable positions throughout the session. Next, the TMS intensity resulted in an MEP amplitude of 1 mV (SI1mV) being determined, and 30 single‐pulse MEPs were recorded (baseline 1, BL1). Following that, RMT and then AMT were obtained. A 10–15 min break was taken after the AMT recording to prevent an effect of muscle contraction on the subsequent measurements. After the break, SICI–ICF, I‐wave facilitation, and the I–O curve were recorded as the first baseline. Subsequently, participants received the pharmacological intervention. A second baseline (BL2), starting with single‐pulse MEP at S1mV, was recorded 2 h after substance intake. If the MEP amplitude deviated more than 20% from baseline 1, TMS intensity was readjusted to achieve an average 1 mV MEP, and baseline three (BL3) was recorded. Next, SICI–ICF, I‐wave facilitation, and the I–O curve were recorded to monitor the influence of the substance on cortical excitability. tDCS was then applied, followed by the TMS protocols immediately and 1 h after plasticity induction. At the end of each session, participants reported any adverse effects of tDCS (Brunoni et al. 2011) and gave verbal feedback on potential side effects of the substance.

### 2.6 Statistical Analysis

All statistical analyses were conducted using IBM SPSS Statistics version 28.0, and the graphs were created using GraphPad Prism version 9.1.0. Mauchly's test of sphericity was conducted for all ANOVAs, and when necessary, the Greenhouse–Geisser correction was applied. Post hoc comparisons were conducted using Fisher's LSD test for exploratory purposes when significant effects were identified in the ANOVA. MEP amplitudes at each time point and for all TMS conditions were averaged for each subject per condition. For the TMS protocols with a double‐pulse condition (i.e., SICI–ICF, I‐wave facilitation), the resulting mean values were normalized to the respective single‐pulse condition. For each ISI, the quotient of the MEP evoked by the conditioning stimulus + test stimulus and the test stimulus alone was calculated. Then, mean values were calculated individually for each time window, and inter‐individual means were calculated for each condition. For the I–O curves, the means of the absolute MEP values were used.

#### 2.6.1 A Priori Differences

To account for any difference in baseline assessments (pre‐medication and pre‐tDCS) that could influence the outcome measures, we conducted one‐way ANOVAs for TMS intensities (SI1 mV) and single‐pulse MEP amplitudes for BL1 (pre‐medication) as well as BL2/3 (pre‐tDCS) separately. Pre‐medication AMTs and RMTs were also analyzed across all sessions. Condition was used as a within‐subject factor. Additionally, for TMS measurements including I–O curve, SICI–ICF, and I‐wave facilitation, two‐way ANOVAs were calculated at pre‐medication and also pre‐tDCS, with condition and ISI or TMS intensity for the I–O curve protocol as within‐subject factors.

#### 2.6.2 Substance Effect

To test if substance alone affected cortical excitability, three‐factorial ANOVAs were calculated with substance (four levels), polarity (two levels), and time (two levels, before and after substance intake) as within‐subject factors for MEP (BL1 vs. BL2) and SI1mV values (BL1 vs. BL3) as dependent variables. Further, we performed repeated‐measures ANOVAs comparing baseline 1 (before substance) and baseline 2 (after substance) for the I–O curve, SICI–ICF, and I‐wave facilitation. For these TMS protocols, substance, polarity, time, and ISI served as within‐subject factors, and MEP amplitudes served as the dependent variable. For the I–O curve, intensity instead of ISI served as within‐subject factor.

#### 2.6.3 Plasticity‐Modulating Effect of all Conditions

To explore if the intervention combinations of substance and tDCS had distinct effects on the TMS outcome measurements (I–O curve, SICI–ICF, and I‐wave facilitation), repeated measures ANOVAs were performed with substance (PLC, l‐dopa, bromocriptine, and sulpiride + l‐dopa), polarity (anodal or cathodal tDCS), time (pre‐stimulation, post‐stimulation 1, and post‐stimulation 2) and ISIs or TMS intensity (I–O curve only) as within‐subject factors, and normalized MEP amplitude (except for the I–O curve, where raw MEP data were used) as the dependent variable to test for statistical significance. Additionally, in case of significant ANOVA results, Fisher's LSD tests were applied to compare each condition after tDCS with its corresponding baseline 2 (pre‐tDCS). Furthermore, a distinct set of Fisher's LSD tests was conducted to assess differences between each DAergic substant condition and the corresponding PLC condition for each time point.

#### 2.6.4 Side Effects

One‐way repeated‐measure ANOVAs were used for each reported tDCS side effect to analyze the presence of adverse effects as a result of tDCS during stimulation, with condition as a within‐subject factor.

## 3 Results

### 3.1 Side Effects

Three to five hours after bromocriptine intake, three participants experienced side effects like dizziness, fatigue, nausea, vomiting, and gastrointestinal discomfort. These effects appeared once in two participants and twice in one, fading within a few hours. Nonetheless, no participant dropped out because of the adverse effects of medications (Table S1a). The average reported side effects of tDCS are summarized in Table S1b. No significant difference was observed between the side effects reported in each condition (Table S2).

### 3.2 A Priori Differences

There were no significant differences in single‐pulse MEP amplitudes, SI1mV, and TMS protocols (I‐O curve, SICI, ICF, and I‐wave facilitation) in the pre‐medication and pre‐tDCS conditions and AMT and RMT in the pre‐medication condition across all sessions; see Supporting Information Material for more details (Tables S3–S7).

### 3.3 Medication Effects Alone

The repeated‐measures two‐way ANOVA showed no significant main effects or interactions for the single‐pulse MEP amplitudes and SI1 mV between baseline 1 and 2/3 (Tables S8 and S9). For the I–O curve protocol, the respective repeated‐measures ANOVA was significant only for the main effect of intensity (*F* = 521.047, df = 1.433, *p* < 0.001) (Table S10). For the SICI–ICF protocol, the main effect of ISI (*F* = 193.164, df = 1.439, *p* < 0.001) and the substance × time interaction (*F* = 5.104, df = 2.275, *p* = 0.008) were significant. Fisher's LSD tests showed a significant difference only for the bromocriptine substance. Bromocriptine increased SICI at ISI 2 ms and decreased ICF at ISIs 10 and 15 ms post‐substance (Table S11, Figure S1). Lastly, the respective ANOVA showed only a significant main effect of ISI (*F* = 46.106, df = 1.623, *p* < 0.001) in the I‐wave facilitation protocol (Table S12). Taken together, the substance only affected SICI–ICF with bromocriptine, while other measurements remained unchanged before tDCS.

### 3.4 Plasticity‐Modulating Effects of Dopaminergic Substances on the I–O Curve

**FIGURE 2:**
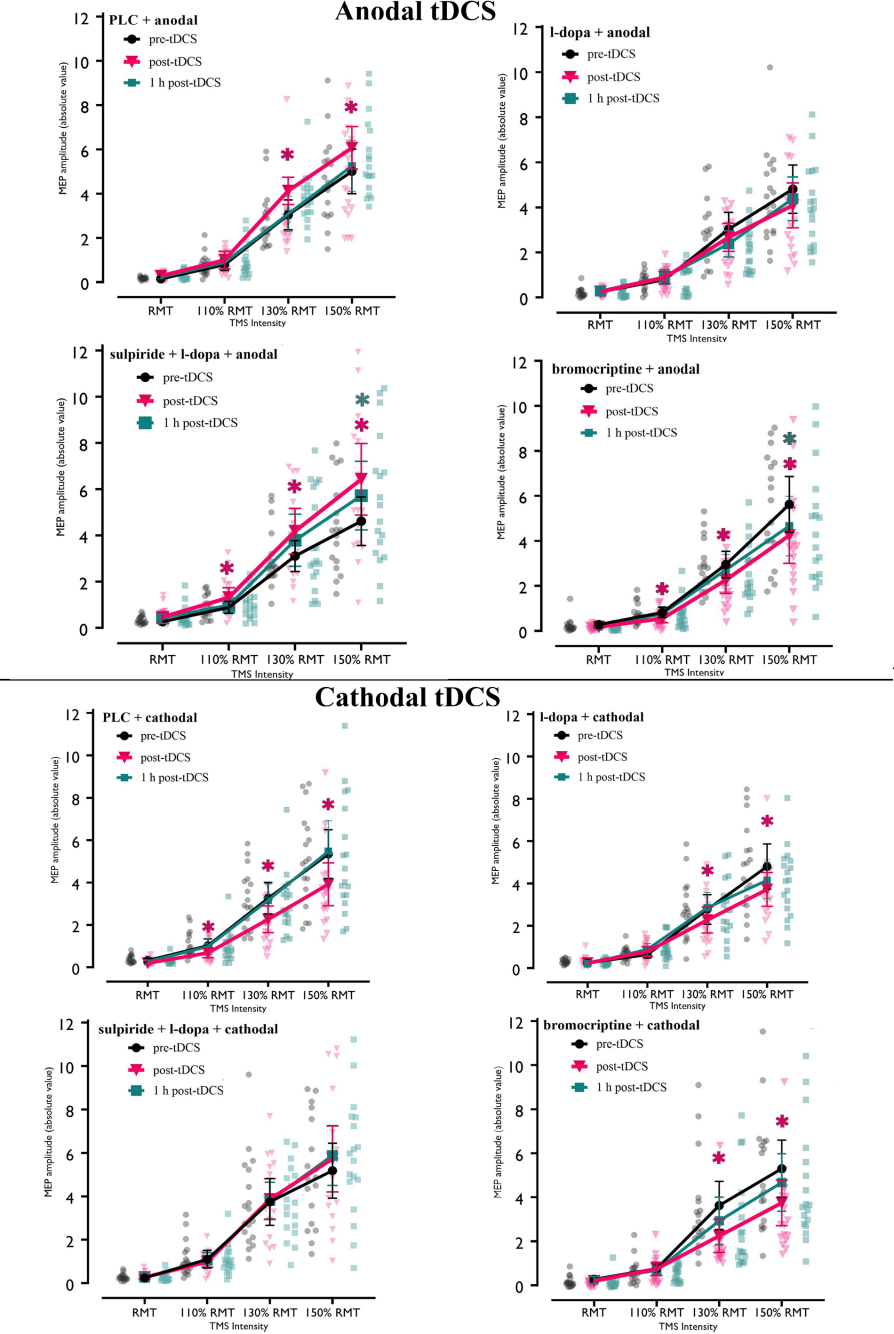
Input–output curve (I–O curve) results for all conditions compared with the respective pre‐tDCS baseline. In the PLC condition, anodal tDCS increased MEP amplitudes at 130% and 150% RMT, while cathodal tDCS decreased them at 110%, 130%, and 150% RMT. With l‐dopa, anodal tDCS had no effect, but cathodal tDCS reduced MEP amplitudes at 130% and 150% RMT. Under bromocriptine, both types of tDCS decreased the I‐O curve. With sulpiride and l‐dopa combined, anodal tDCS increased the I–O curve, while cathodal tDCS had no effect. Error bars represent the 95% confidence interval. Background dots display the distribution of individual data points. Magenta and green asterisks (*) indicate significant differences between pre‐ and immediately post‐stimulation and pre‐ and 1‐h post‐stimulation time windows, respectively, based on Fisher's LSD post hoc comparisons. PLC, placebo.

The results of the repeated‐measures ANOVA revealed significant main effects of intensity and substance, and significant interactions of substance × time, polarity × time, substance × polarity × time, substance × time × intensity, and polarity × time × intensity. Other main effects and interactions were not significant (Table 1). For post hoc analysis, we compared post‐tDCS results with baseline 2 (pre‐tDCS). In the PLC condition, Fisher's LSD tests showed significantly larger MEP amplitudes after anodal tDCS at TMS intensities of 130% and 150% RMT immediately post‐stimulation and decreased MEP amplitudes at 110%, 130%, and 150% RMT immediately after cathodal tDCS, as compared to baseline (Figure 2). Under l‐dopa, anodal tDCS did not change the I–O curve, but cathodal tDCS reduced MEP amplitudes at 130% and 150% RMT compared to baseline immediately post‐stimulation (Figure 2). Under bromocriptine and anodal tDCS, MEP amplitudes were reduced at 110%, 130%, and 150% RMT immediately post‐stimulation and at 150% RMT at the second time point after stimulation. MEP amplitudes also significantly decreased at 130% and 150% RMT immediately after cathodal tDCS (Figure 2). Finally, under concurrent administration of sulpiride and l‐dopa, anodal tDCS increased MEP amplitudes at 110% and 130% RMT immediately post‐stimulation and at 150% RMT up to the second time point post‐stimulation. Under sulpiride and l‐dopa combined, cathodal tDCS did not alter the I‐O curve as compared to the baseline (for an overview see Figure 2).

**TABLE 1:** Results of the repeated measures ANOVA conducted for the I–O curve.

Factor	df	*F*	*p*	*η* ^2^_*p*_
Substance	1.872^a^	4.123	**0.027** *	0.195
Polarity	1	0.225	0.641	0.013
Time	2	1.269	0.294	0.069
Intensity	1.323^a^	473.687	**< 0.001** *	0.965
Substance × Polarity	2.791^a^	0.179	0.898	0.010
Substance × Time	4.433^a^	7.324	**< 0.001** *	0.301
Polarity × Time	1.948^a^	11.445	**< 0.001** *	0.402
Substance × Polarity × Time	4.037^a^	2.584	**0.044** *	0.131
Substance × Intensity	2.292^a^	2.524	0.086	0.129
Polarity × Intensity	1.219^a^	0.327	0.616	0.018
Substance × Polarity × Intensity	3.431^a^	0.373	0.798	0.021
Time × Intensity	2.973^a^	1.580	0.205	0.085
Substance × Time × Intensity	5.817^a^	4.709	**< 0.001** *	0.216
Polarity × Time × Intensity	3.200^a^	5.933	**0.001** *	0.258
Substance × Polarity × Time × Intensity	7.416^a^	1.283	0.261	0.070

Abbreviations: df = degrees of freedom, *η* ^2^_*p*_ = partial eta squared.

^a^ Greenhouse–Geisser correction according to violation of sphericity.

* *p* < 0.05.

**FIGURE 3:**
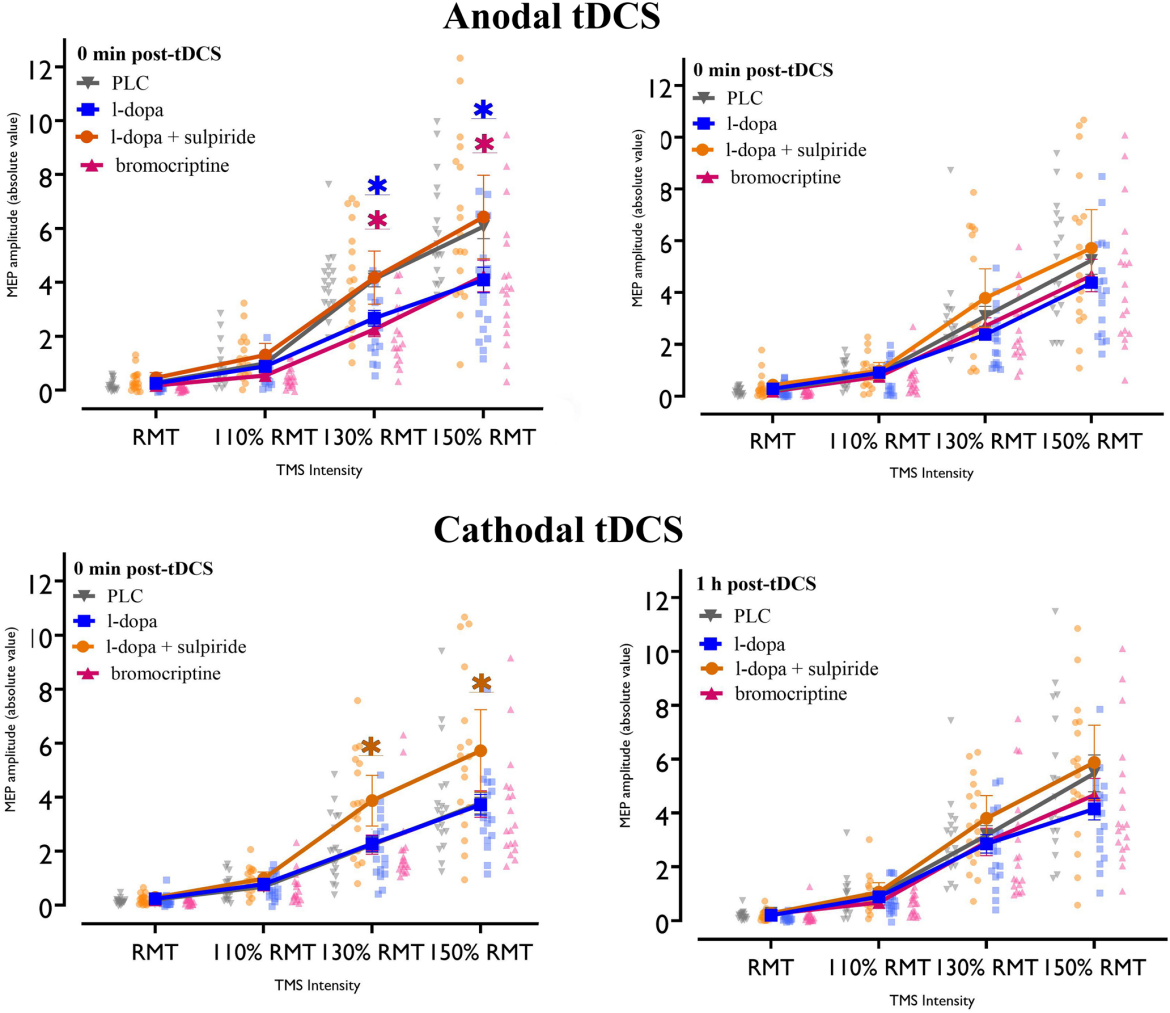
Input–output curve (I–O curve) results for all conditions after stimulation compared to PLC. L‐dopa combined with anodal tDCS decreased the I‐O curve at 130% and 150% RMT, while l‐dopa with cathodal tDCS did not affect MEP amplitudes compared to PLC. Bromocriptine with anodal tDCS reduced MEP amplitudes at 130% and 150% RMT, but no difference was observed with bromocriptine and cathodal tDCS. Anodal tDCS with sulpiride and l‐dopa showed no MEP changes, while cathodal tDCS significantly enhanced MEP amplitudes at 130% and 150% RMT compared to PLC. Error bars represent the 95% confidence interval. Background dots display the distribution of individual data points. Blue, pink, and orange asterisks (*) indicate significant differences between l‐dopa, bromocriptine, and sulpiride + l‐dopa vs. the PLC condition (in gray) respectively, based on Fisher's LSD post hoc comparisons. PLC, placebo.

We also compared each DAergic substance with its respective placebo (PLC + anodal or PLC + cathodal). Under l‐dopa, post hoc comparisons showed a significant reduction of MEPs at 130% and 150% immediately after anodal tDCS, while the I–O curve after cathodal tDCS was not altered compared to PLC (Figure 3). Bromocriptine compared to PLC caused a significant reduction of the MEP amplitudes at 130% and 150% RMT for anodal tDCS but did not affect the I–O curve after cathodal tDCS (Figure 3). Finally, whereas combined sulpiride and l‐dopa did not affect the I–O curve after anodal tDCS, a significant enhancement of MEP amplitudes at 130% and 150% RMT was observed immediately after cathodal tDCS as compared to the PLC condition (for an overview see Figure 3).

### 3.5 Plasticity‐Modulating Effects of Dopaminergic Substance on SICI–ICF

**FIGURE 4:**
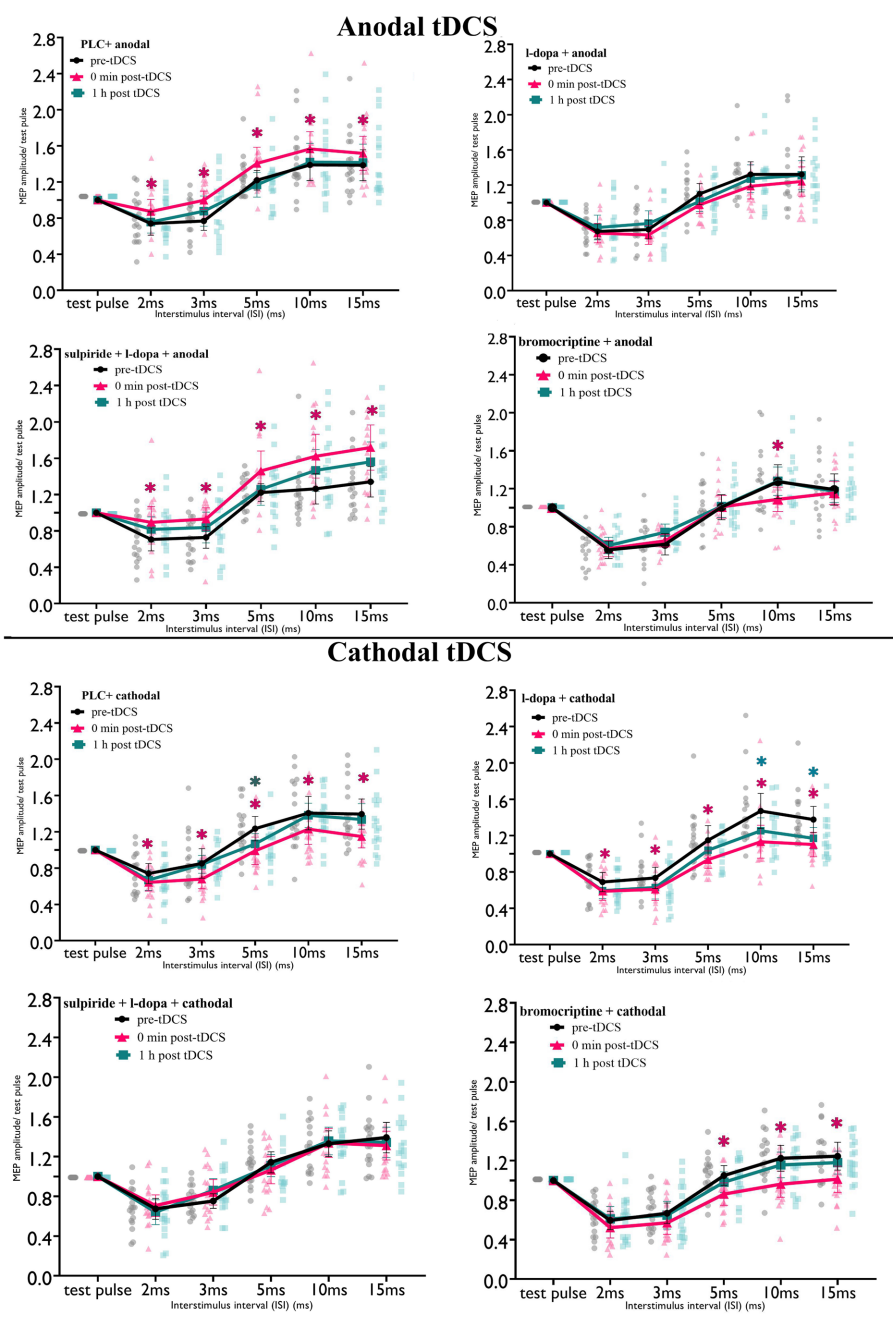
Short‐interval intracortical inhibition (SICI) and facilitation (ICF) results for all conditions compared to the respective pre‐tDCS baseline. Under PLC, anodal tDCS decreased SICI and increased ICF, while cathodal tDCS had the opposite effect. With l‐dopa, anodal tDCS had no effect, but cathodal tDCS increased SICI and decreased ICF. Anodal tDCS with bromocriptine decreased ICF at a 10 ms ISI, and cathodal tDCS also reduced ICF. With l‐dopa + sulpiride, anodal tDCS reduced SICI and enhanced ICF, while cathodal tDCS effects were abolished. Error bars represent the 95% confidence interval. Background dots display the distribution of individual data points. Magenta and green asterisks (*) indicate significant differences between pre‐ and immediately post‐stimulation and pre‐ and 1‐h post‐stimulation, respectively, based on Fisher's LSD post hoc comparisons. PLC, placebo.

The repeated‐measures ANOVA showed significant main effects of substance, polarity, and ISI, and significant interactions for substance × time, polarity × time, substance × polarity × time, substance × ISI, time × ISI, substance × time × ISI, and polarity × time × ISI. Other main effects and interactions were not significant (Table 2). Compared to the respective baseline (pre‐tDCS) under PLC, post hoc comparisons showed that anodal tDCS significantly decreased SICI and increased ICF at all ISIs immediately post‐stimulation (Figure 4). Under l‐dopa combined with anodal tDCS, SICI and ICF remained unchanged, while cathodal tDCS combined with l‐dopa significantly increased SICI at all ISIs immediately post‐stimulation and decreased ICF at all ISIs up to the second time point post‐stimulation compared to baseline (Figure 4). Under bromocriptine, anodal tDCS did not affect SICI but reduced ICF at ISI 10 immediately post‐stimulation, while cathodal tDCS combined with bromocriptine decreased ICF at all ISIs immediately post‐stimulation (Figure 4). When combined sulpiride and l‐dopa were administered, anodal tDCS decreased SICI and increased ICF compared to baseline immediately post‐stimulation, while cathodal tDCS did not alter SICI and ICF under these substances (for an overview see Figure 4).

**TABLE 2:** Results of the repeated measures ANOVA for SICI–ICF.

Factor	df	*F*	*p*	*η* ^2^_*p*_
Substance	3	15.125	**< 0.001** *	0.470
Polarity	1	6.569	**0.020** *	0.278
Time	2	1.764	0.186	0.094
ISI	1.705^a^	235.701	**< 0.001** *	0.932
Substance × Polarity	3	1.706	0.177	0.091
Substance× Time	3.977^a^	6.846	**< 0.001** *	0.287
Polarity × Time	1.385^a^	10.120	**< 0.001** *	0.373
Substance× Polarity × Time	6	4.449	**< 0.001** *	0.207
Substance × ISI	5.859^a^	3.080	**0.008** *	0.153
Polarity × ISI	2.069^a^	1.772	0.183	0.094
Substance× Polarity× ISI	15	0.930	0.531	0.051
Time× ISI	10	2.719	**0.004** *	0.137
Substance × Time× ISI	10.015^a^	2.346	**0.012** *	0.121
Polarity × Time × ISI	10	3.357	**< 0.001** *	0.164
Substance × Polarity × Time × ISI	9.742^a^	1.810	0.064	0.096

Abbreviations: df = degrees of freedom, *η* ^2^_*p*_ = partial eta squared.

^a^ Greenhouse–Geisser correction according to violation of sphericity.

* *p* < 0.05.

**FIGURE 5:**
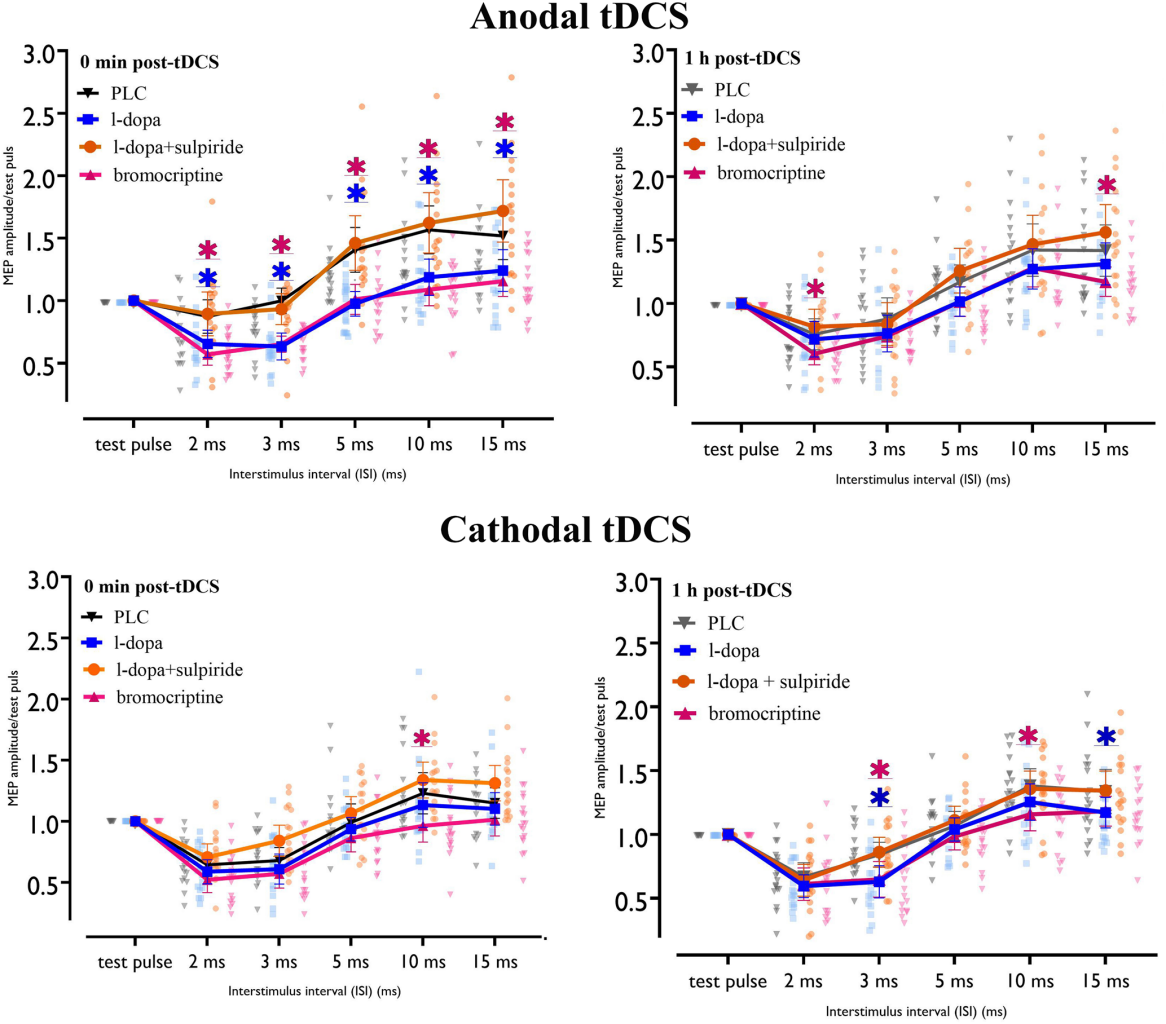
Short‐interval intracortical inhibition (SICI) and facilitation (ICF) for all conditions after stimulation compared to PLC. L‐dopa with anodal tDCS increased SICI and decreased ICF compared to PLC. L‐dopa and cathodal tDCS enhanced SICI (3 ms ISI) and reduced ICF (15 ms ISI) at 1 h post‐tDCS. Bromocriptine with anodal tDCS increased SICI and decreased ICF up to 1 h post‐tDCS. Bromocriptine with cathodal tDCS reduced ICF (10 ms ISI) at both time points and enhanced SICI (3 ms ISI) at the second time point. Sulpiride with l‐dopa and both tDCS types showed no differences compared to PLC. Error bars represent the 95% confidence interval. Background dots display the distribution of individual data points. Blue, pink, and orange asterisks (*) indicate significant differences between l‐dopa, bromocriptine, and sulpiride + l‐dopa versus the PLC condition (in black) respectively based on Fisher's LSD post hoc comparisons. PLC, placebo.

Post hoc comparisons showed increased SICI and reduced ICF at all ISIs immediately post‐stimulation in the l‐dopa with anodal tDCS condition, compared to placebo. Under l‐dopa and cathodal tDCS, SICI at ISI 3 ms was enhanced, and ICF at ISI 10 was decreased up to 1 h post‐stimulation (Figure 5). Bromocritine combined with anodal tDCS, compared to placebo, showed increased SICI and decreased ICF at all ISIs at the first time point, with these effects persisting for ISIs 2 and 15 until the second post‐stimulation time point. Bromocriptine combined with cathodal tDCS compared to PLC decreased ICF at ISI 10 for both the first and second time points after stimulation and enhanced SICI at ISI 3 at the second time point (Figure 5). Lastly, sulpiride plus l‐dopa combined with both anodal and cathodal tDCS did not significantly differ from the respective PLC conditions (for an overview see Figure 5).

### 3.6 Plasticity‐Modulating Effects of Dopaminergic Substance on I‐Wave Facilitation

The repeated measures ANOVA indicated that there were no significant main effects or interaction effects associated with the factor medication (for an overview see Table S13).

## 4 Discussion

This study investigated the contribution of glutamatergic and GABAergic mechanisms to the tDCS‐generated plasticity‐modulating effects of DA in the human motor cortex. We used TMS protocols that measure intracortical (SICI, ICF, and I‐waves) and corticospinal (I–O curve) excitability for monitoring glutamatergic and GABAergic activities, and explored the impact of global dopaminergic, D2‐like, and D1‐like receptor activation on tDCS‐induced LTP‐ and LTD‐like plasticity. Corticospinal excitability measurements and the I‐wave facilitation protocol revealed no effect of the substances alone on these parameters. For SICI/ICF, only the D2 receptor agonist bromocriptine enhanced intracortical inhibition and reduced facilitation. Our main findings indicate that DA itself, as a neuromodulator, showed no major direct effects on motor cortex excitability, except for bromocriptine. However, the significant neurophysiological changes observed following tDCS administration under pharmacological intervention were specifically linked to the interaction between general DA, D1‐ and D2‐like receptor modulation, and tDCS polarity, as these effects could not be fully explained by the substance or tDCS alone. Significant alterations of glutamate‐dependent SICI–ICF and the I–O curve suggest that the modulation of glutamatergic mechanisms specifically mediated by the DA receptor subtypes underlies the impact of DA on tDCS‐induced plasticity, as described below. The findings indicate that the effects of DA and D2 activation on tDCS‐induced plasticity are associated with decreased corticospinal and intracortical excitability, while D1‐like receptor activation affected tDCS‐induced plasticity by increasing facilitation and decreasing inhibition. In the following sections, we discuss in detail the effects of dopaminergic substances, tDCS, and their combination on cortical excitability and propose mechanistic explanations for the observed effects.

### 4.1 Effects of Interventions on Corticospinal Excitability

Our results revealed that global DA, D1, and D2 receptor activation alone did not alter single‐pulse MEPs and the I–O curve, which confirms the results of previous studies (Fresnoza, Paulus, et al. 2014; Fresnoza, Stiksrud, et al. 2014; Kuo et al. 2007; Monte‐Silva et al. 2010). When we applied tDCS alone, anodal and cathodal tDCS increased and decreased the I–O curves, respectively, which is in line with a previous study (Nitsche et al. 2005). Regarding the dopaminergic impact on tDCS‐induced plasticity, global DA and D2‐like receptor activation abolished the facilitatory effects of anodal tDCS on the I–O curve while preserving the LTD‐like effects induced by cathodal tDCS. In contrast, D1‐like receptors preserved the enhancement of the I–O curve induced by anodal tDCS and abolished the effects of cathodal tDCS. These results are again compatible with previous studies about the plasticity‐modulating effects of dopaminergic interventions (Fresnoza, Paulus, et al. 2014; Fresnoza, Stiksrud, et al. 2014; Ghanavati et al. 2022; Monte‐Silva et al. 2010).

### 4.2 Effects of Interventions on Intracortical Excitability

Pre‐intervention SICI and ICF were not modified by general DA activation and D1‐like activation, which is in line with previous studies (Martorana et al. 2008; Ziemann et al. 1997). D2 activation by bromocriptine, however, increased SICI and decreased ICF, also in line with previous studies showing increased SICI and reduced ICF by bromocriptine and enhanced SICI by the D2/D3 agonist cabergoline (Korchounov et al. 2007; Ziemann et al. 1997).

Under the PLC condition, anodal tDCS increased ICF and decreased SICI, while cathodal tDCS had the opposite effect, consistent with previous studies on tDCS effects on SICI and ICF (Melo et al. 2024; Nitsche et al. 2005). Concerning combined substance and tDCS interventions, Global DAergic and D2‐like receptor activation reduced cortical excitability from anodal tDCS, enhancing SICI and reducing ICF. Cathodal tDCS effects, including SICI enhancement and ICF reduction, were preserved and prolonged by this activation. These results align with previous findings, where global DAergic and D2‐like receptor activation reversed anodal tDCS‐induced plasticity enhancement and prolonged cathodal tDCS after‐effects (Fresnoza, Stiksrud, et al. 2014; Fresnoza et al. 2021; Ghanavati et al. 2022; Kuo et al. 2007). Furthermore, our findings suggest that under D1‐like receptor activation, the anodal tDCS‐induced SICI reduction and ICF enhancement were preserved, while the cathodal tDCS‐generated SICI enhancement and ICF reduction were not observed, which is also in line with the results of previous single‐pulse TMS studies (Fresnoza, Paulus, et al. 2014; Nitsche et al. 2009).

Regarding I‐wave facilitation, DAergic substances alone did not affect cortical excitability. Anodal tDCS increased I‐wave facilitation, while cathodal tDCS slightly reduced it, which is partially consistent with prior research (Nitsche et al. 2005). DAergic substances combined with tDCS, however, showed no significant effect on I‐wave facilitation.

### 4.3 Proposed Mechanisms

The impact of DAergic activation on glutamate/NMDA receptors is the likely mechanism underlying modulatory effects of general DA activation, D1‐, and D2‐like receptors on tDCS‐induced plasticity, as indicated by substance‐dependent changes after stimulation in the I‐O curve and SICI–ICF, which are influenced by the glutamatergic system (Ziemann, Chen, et al. 1998). However, this intervention did not affect I‐wave facilitation, which is regulated by the GABAergic system, but not the glutamatergic system (Chen 2000). The I–O curve is reduced by sodium and calcium channel blockers and modulated by substances affecting the glutamatergic system at higher TMS intensities (Paulus et al. 2008). In accordance with the contribution of the glutamatergic system to the DA‐dependent alterations of tDCS‐induced plasticity, we see the main effects of the substances at higher TMS intensities. In further accordance, dopaminergic modulation of tDCS‐induced plasticity had a prominent impact on SICI and ICF. Glutamate is the main regulator of ICF since it is reduced by the NMDA blocker dextromethorphan (Ziemann, Chen, et al. 1998), while SICI is enhanced by GABA receptor agonists and by NMDA receptor antagonists (Dahlhaus et al. 2008; Ziemann, Chen, et al. 1998; Ziemann et al. 1996b), and is thus affected by the glutamatergic and GABAergic systems. Although the results show DA's effect on tDCS‐induced plasticity involves the glutamatergic system, a contribution from the GABAergic system cannot be excluded. However, it seems unlikely since the exclusively GABA‐controlled I‐wave facilitation (Ziemann, Chen, et al. 1998; Ziemann, Tergau, et al. 1998) after tDCS was not affected by dopaminergic substances. The proposed mechanism is supported by tDCS‐induced plasticity being mainly NMDA/glutamate‐dependent, as shown by the plasticity‐abolishing effect of NMDA antagonists, and a beneficial effect of an NMDA receptor agonist which also restored anodal tDCS‐induced plasticity originally blocked by global DA and D2‐like receptor activation (Ghanavati et al. 2022; Nitsche, Fricke, et al. 2003; Nitsche, Jaussi, et al. 2004). In contrast, GABA enhancement had no prominent effect on motor cortical excitability alterations induced by tDCS (Nitsche, Liebetanz, et al. 2004), and an MRS study showed a polarity‐unspecific effect of tDCS on GABA, namely reduced GABA concentration (Stagg et al. 2009). Therefore, Our findings suggest that glutamate plays a more critical role than non‐specific GABA effects in dopaminergic modulation of tDCS‐induced plasticity.

For LTP‐like plasticity, our results suggest a common mechanism of global DA and D2‐like receptors, indicated by a decreased or absent excitability‐enhancing effect of anodal tDCS on SICI–ICF and the I–O curve, in line with previous works (Fresnoza, Stiksrud, et al. 2014; Ghanavati et al. 2022; Monte‐Silva et al. 2010). It is suggested that D2 receptors have a suppressing effect on NMDA receptor activity (Higley and Sabatini 2010). This would counteract the anodal tDCS‐induced NMDA receptor activity enhancement, thus blocking LTP‐like plasticity, and lead to reduced ICF and corticospinal excitability, as well as enhanced intracortical inhibition, which involves the glutamatergic system (Paulus et al. 2008; Ziemann, Chen, et al. 1998). For the global DA effect, the relatively low‐level diffuse activity induced by tDCS might not be sufficient to activate D1‐like receptors to the amount required to counterbalance the NMDA receptor activity‐reducing effect of D2 receptors (Ghanavati et al. 2022; Kuo et al. 2007). Moreover, D2 receptors are more frequent than D1 receptors in the motor cortex (Hosp and Luft 2013; Papenberg et al. 2019).

For LTD‐like plasticity, the cathodal tDCS effects on cortical excitability were prolonged (reduced ICF and enhanced SICI) or preserved (reduction of I–O curve amplitudes) by global DA activation, and the D2‐like receptor agonist. The proposed mechanisms of action for LTD‐like plasticity are similar to the impact of general DA and D2‐like receptor activation on LTP‐like plasticity because LTD‐like plasticity induced by cathodal tDCS also depends on the glutamatergic system (Liebetanz et al. 2002). The prolonging effect of global dopaminergic and D2‐like receptor activation on LTD‐like plasticity is likely due to D2 receptor‐dependent stabilization of this plasticity (Otani et al. 1998; Seamans and Yang 2004), as D2 receptors gradually decrease NMDA receptor activity, likely optimizing it for LTD‐like plasticity induction. These results align with animal studies showing that DA application to the frontal cortex decreases neuronal excitability and promotes LTD over LTP in local glutamatergic synapses (Gulledge and Jaffe 1998; Otani et al. 1998). Additionally, D2 receptor stimulation has been shown to induce LTD of NMDA receptor‐mediated components in PFC slices and convert LTP into LTD in the striatum (Banks et al. 2015; Tseng and O'Donnell 2004). Furthermore, both general DA and D2‐like receptor activation have been shown to impair LTP‐related learning processes in humans and non‐human primates (Marino and Levy 2019; Vo et al. 2017).

In contrast to D2 receptor activation, D1 receptors preserved the decreased SICI, enhanced ICF, and increased the I–O curve generated by anodal tDCS. For LTD‐like plasticity, D1 receptor activation enhanced ICF and the recruitment curve and reduced SICI but had no impact on the effects of tDCS on I‐wave facilitation, again in accordance with a primary impact of the glutamatergic system. This is in line with previous studies, where anodal tDCS after‐effects were preserved while neuroplastic cathodal tDCS effects were abolished by D1‐like receptor activation (Fresnoza, Chen, et al. 2014; Nitsche et al. 2009) This pattern of results is furthermore supported by animal experiments showing that D1‐like receptors enhance NMDA receptor activity, which in turn facilitates synaptic LTP‐like plasticity and long‐term memory formation (Li et al. 2003, 2010; Seamans and Yang 2004). Our results suggest that D1 receptor activation shifts the cortical excitation‐inhibition balance toward excitation, with the LTD‐like plasticity reduction likely due to enhanced NMDA receptor activity. These results mirror animal studies where D1‐like activation converted LTD to LTP and enhanced synaptic efficacy (Gurden et al. 2000; Mockett et al. 2007; Schmalz and Kumar 2022). The results of our study are in line with the role of D1 receptors in learning and memory formation by enhancing cortical excitability and LTP‐like plasticity, as suggested by animal and human experiments (Chen et al. 2020; Rossato et al. 2009; Schmalz and Kumar 2022; Seamans et al. 1998).

### 4.4 Functional and Clinical Implications

Our findings suggest that D1 receptor activation shifts cortical plasticity towards excitation and LTP‐like plasticity, while D2 receptor activation promotes inhibition and LTD‐like plasticity (Fresnoza, Chen, et al. 2014; Fresnoza, Stiksrud, et al. 2014; Ghanavati et al. 2022; Kuo et al. 2007). These findings have implications for brain stimulation interventions targeting plasticity deficits in neuropsychiatric and neurological disorders, particularly those with DA dysregulation such as schizophrenia, Parkinson's disease, OCD, and ADHD, which showed positive responses to tDCS and other noninvasive brain stimulation interventions (Li et al. 2024; Pol et al. 2021; Salehinejad, Ghanavati, 2024; Glinski, et al. 2022). Understanding how subtype‐specific DA receptors contribute to LTP‐ and LTD‐like plasticity can inform treatment strategies for these disorders (Bhandari et al. 2016; Mishra et al. 2018). Our findings suggest a clinical potential of targeting D1 receptors for enhancing motor functions and addressing cognitive impairments in stroke and Parkinson's disease. In contrast, l‐dopa and D2/D3 agonists showed no efficacy in improving motor function in individuals with chronic stroke (Cramer et al. 2009; Restemeyer et al. 2007). Similarly, dopaminergic agents that have not only D2‐ but also D1 receptor‐enhancing effects might have advantages for improving motor impairments in Parkinson's disease (Brusa et al. 2005; Mailman et al. 2021). Another clinical implication could be the potential anti‐epileptic efficacy of combining D2 agonists with cathodal tDCS. Cathodal tDCS reduces seizures (Fregni et al. 2006; Yang et al. 2019), and D2 receptor activation, which may downregulate glutamate, could enhance this effect. Bromocriptine, a D2 agonist, has already shown effectiveness in reducing seizure frequency, suggesting a synergistic approach with non‐invasive brain stimulation (Bozzi and Borrelli 2013).

### 4.5 Limitations

One limitation of this study is the small sample size; thus, the obtained results should be replicated in future studies. Moreover, our sample size includes healthy young participants, and the findings cannot be directly transferred to other populations, including patients. The serum concentration of the substances was not determined in the present study, which might have delivered information about dosage‐dependent effects, which are relevant for dopaminergic substances (Pearson‐Fuhrhop et al. 2013; Witte et al. 2012). A further limitation is that the primary outcome measure was the relatively unspecific ANOVA, while we chose liberal exploratory post hoc tests based on the ANOVA results for more specific analyses, to keep this study feasible. Therefore, the results should be regarded as preliminary and substantiated by follow‐up studies with a larger sample size. Another limitation of this study is the lack of systematic assessment of participants' family history of Parkinson's disease or the presence of REM sleep behavior disorder (RBD), which is a prodromal marker of Parkinsonism and may be associated with early dopaminergic changes. Future research should account for these variables to better control for variability or explore them systematically. Finally, our finding regarding DAergic modulation of plasticity was based on indirect TMS measurements. Future studies might directly assess relevant neurotransmitter systems in animal models or by magnetic resonance spectroscopy or positron emission tomography in humans.

### 4.6 Conclusion

The results of the present study show specific receptor subtype‐dependent effects of dopaminergic activation on tDCS‐induced plasticity. General DA and D2 receptor activation reduced LTP‐like plasticity and preserved LTD‐like plasticity. This was linked to decreased corticospinal and ICF and increased intracortical inhibition. Conversely, D1 receptor activation preserved and abolished LTP and LTD‐like plasticity, respectively, associated with enhanced corticospinal and ICF and decreased intracortical inhibition. The specific changes in TMS parameters indicate the important role of the glutamatergic system in modulating the effects of DA on plasticity.

## Author Contributions

E.G. conducted the experiment, collected and analyzed the data, wrote the paper, and designed illustrations. M.A.S. contributed to data analysis, illustrations and revised the work. M.C.B. contributed to data collection, literature searches, and drafting. L.M. contributed to data analysis and handled blinding and allocation concealment. A.F. contributed to data analysis and illustrations. M.‐F.K. and M.A.N. conceived and designed the experiments and critically reviewed the final draft. All authors contributed to the final drafting of the work.

## Conflicts of Interest

M.A. Nitsche is a member of the Scientific Advisory Boards of Neuroelectrics, Precisis, and served as a scientific consultant for Boehringer Ingelheim. The other authors declare no conflicts of interest.

## Supporting information


**Data S1.** Supporting Information.

## Data Availability

The data that support the findings of this study are available on request from the corresponding author. The data are not publicly available due to privacy or ethical restrictions.
