# Supplementary material for: Contribution of Glutamatergic and GABAergic Mechanisms to the Plasticity‐Modulating Effects of Dopamine in the Human Motor Cortex
Source: Hum Brain Mapp. 2025 Feb 13;46(3):e70162. doi: 10.1002/hbm.70162 (PMC11822652; doi:10.1002/hbm.70162)
Supplement: Supplementary file 1 — Data S1. Supporting Information. [file HBM-46-e70162-s001.docx]

***Supplementary material***

**Table S1a**: Reported side effects associated with substance given in the session.

| **Participant** | **Substance given in the respective session (symptoms)** |
| --- | --- |
| A | bromocriptine (nausea, dizziness, vomiting) bromocriptine (nausea, fatigue) |
| B | bromocriptine (nausea, gastrointestinal discomfort) |
| C | bromocriptine (nausea, dizziness) |

**Table S1b**: Reported side effects of tDCS during stimulation under each substance.

| Side effect | **Condition** | | | |
| --- | --- | --- | --- | --- |
|  | **PLC + cathodal** | **PLC + anodal** | **l-dopa + cathodal** | **l-dopa + anodal** |
| **Visual** | 0.055 ± 0.235 | 0.055 ± 0.235 | 0.055 ± 0.235 | 0.00 |
| **Itching** | 1.555 ± 0.921 | 0.777 ± 0.732 | 1.944 ± 0.872 | 1.500 ± 1.200 |
| **Tingling** | 1.722 ± 1.227 | 1.333 ± 0.840 | 1.444 ±1.149 | 1.277 ± 0.894 |
| **Burning** | 1.444 ± 1.041 | 1.111 ± 0.900 | 1.444 ± 1.149 | 1.611 ± 1.036 |
| **Pain** | 0.555 ± 0.921 | 0.444 ± 0.855 | 0.611 ± 0.849 | 0.388 ± 0.978 |
|  | **Condition** | | | |
|  | **sulpiride + l-dopa + cathodal** | **sulpiride + l-dopa + anodal** | **bromocriptine + cathodal** | **bromocriptine + anodal** |
| **Visual** | 0.055 ±0.235 | 0.222±0.427 | 0.00 | 0.00 |
| **Itching** | 1.333 ±1.084 | 1.666 ± 1.236 | 1.277 ± 1.074 | 1.388 ± 1.243 |
| **Tingling** | 1.555 ± 0.921 | 1.555 ± 0.983 | 1.277 ± 1.178 | 0.888 ± 0.758 |
| **Burning** | 1.222 ± 1.060 | 1.611 ± 1.334 | 1.722 ± 1.178 | 1.555 ± 1.199 |
| **Pain** | 0.833 ± 1.043 | 0.277 ± 0.751 | 0.277 ± 0.751 | 0.888 ± 1.231 |

*Note:* The presence and intensity of side effects were rated on a numerical scale ranging from zero to five, with zero representing no and five representing extremely strong sensations. Data are presented as mean ± SD. tDCS = transcranial direct current stimulation; PLC = placebo.

**Table S2**: Repeated-measures ANOVA results for the intensity of reported tDCS side effects during stimulation

| **Side-effects** | **Factors** | **df** | **F Value** | ***P*-Value** | ${}_{\boldsymbol{p}}^{\mathbf{2}}$ |
| --- | --- | --- | --- | --- | --- |
| Visual | Condition | 2.570^#^ | 1.943 | 0.144 | 0.103 |
| Itching | Condition | 3.941^#^ | 1.884 | 0.130 | 0.100 |
| Tingling | Condition | 1.445^#^ | 1.401 | 0.260 | 0.076 |
| Burning | Condition | 7 | 0.718 | 0.718 | 0.041 |
| Pain | Condition | 3.153^#^ | 1.918 | 0.135 | 0.101 |

*Note*: The intensity of reported side effects during tDCS were analyzed by repeated-measure ANOVAs. df = degrees of freedom, η2p = partial eta squared, # Greenhouse−Geisser correction according to violation of sphericity.

**Table S3.** Single-pulse MEP amplitudes and SI1mVpre-substance administration and pre-tDCS (baselines 1, 2 and 3).

| **Condition** | **Corticospinal excitability**  Single-pulse MEP (mV) | | | **TMS intensity**  SI1mV (% MSO) | |
| --- | --- | --- | --- | --- | --- |
|  | **BL1** | **BL2** | **BL3** | **BL1** | **BL3** |
| **anodal + PLC** | 1.01±0.1 | 1.03±0.2 | 1.05±0.07 | 45.5±5.99 | 45.5±6.25 |
| **anodal + l-dopa** | 1.00±0.08 | 1.01±0.26 | 1.00±0.11 | 45.83±6.77 | 45.66±6.66 |
| **anodal + bromocriptine** | 1.01±0.09 | 0.99±0.17 | 1.02±0.08 | 45.22±6.03 | 45.38±5.91 |
| **anodal + sulpiride + l-dopa** | 1.05±0.08 | 1.03±0.24 | 1.03±0.10 | 45.38±6.01 | 45.05±6.22 |
| **cathodal + PLC** | 1.05±0.08 | 1.03±0.17 | 0.97±0.11 | 45.33±6.25 | 45.05±6.37 |
| **cathodal + l-dopa** | 1.02±0.08 | 1.00±0.21 | 0.99±0.13 | 46.05±6.03 | 46±6.24 |
| **cathodal + bromocriptine** | 1.02±0.10 | 0.99±0.24 | 0.99±0.13 | 44.77±6.18 | 44.16±6.29 |
| **cathodal + sulpiride + l-dopa** | 0.98±0.9 | 1.06±0.16 | 1.00±0.09 | 46.05±6.53 | 46.27±6.15 |

Baseline 1 (BL1) refers to the measurements at the beginning of each session. Baseline 2 (BL2) refers to the measurements performed 2 hours after substance intake and before stimulation, and Baseline 3 (BL3) refers to the measurements conducted immediately after BL2, after TMS intensity adjustment, if required. Data are presented as mean ± SD. MEP: motor evoked potentials. SI1mV: stimulation output required to generate ~1mV MEP. % MSO: percentage of maximal stimulator output. mV: millivolts. PLC = placebo.

**Table S4**. Results of one-way ANOVAs for MEP amplitude and SI1 mV, both pre-substance and pre-tDCS, as well as AMT and RMT pre-substance administration.

| **MEP amplitude, pre-substance** | | | | |
| --- | --- | --- | --- | --- |
| **Factor** | **df** | **F value** | **p-value** | **η^2^_p_** |
| Session/ **MEP amplitude, pre-substance** | 7 | 1.195 | 0.311 | 0.066 |
| Session/ **MEP amplitude, pre-tDCS** | 7 | 1.075 | 0.384 | 0.059 |
| Session/ **SI1mV,** **pre-substance** | 2.160^#^ | 1.052 | 0.363 | 0.058 |
| Session/ **SI1mV, pre-tDCS** | 2.318^#^ | 1.249 | 0.301 | 0.068 |
| Session/ **AMT, pre-substance** | 7 | 1.146 | 0.339 | 0.063 |
| Session/ **RMT, pre-substance** | 7 | 0.888 | 0.519 | 0.050 |

df = degrees of freedom, η^2^p = partial eta squared, ^#^ Greenhouse−Geisser correction according to violation of sphericity; *p < 0.05

**Table S5.** Results of the two-way ANOVAs for I-O curve, pre-substance administration and pre-tDCS.

| **I-O curve, pre-substance** | | | | |
| --- | --- | --- | --- | --- |
| **Factor** | **df** | **F value** | **p-value** | **η^2^_p_** |
| Condition | 7 | 0.546 | 0.798 | 0.31 |
| Intensity | 1.389^#^ | 401.314 | **<0.001*** | 0.959 |
| Condition × Intensity | 6.944^#^ | 0.582 | 0.768 | 0.033 |
| **I-O curve, pre-tDCS** |  | | | |
| Condition | 7 | 0.491 | 0.839 | 0.28 |
| Intensity | 1.672^#^ | 435.474 | **<0.001*** | 0.962 |
| Condition × Intensity | 6.050^#^ | 0.634 | 0.779 | 0.036 |

df = degrees of freedom, η^2^p = partial eta squared, ^#^ Greenhouse−Geisser correction according to violation of sphericity; *p < 0.05

**Table S6.** Results of the two-way ANOVAs for SICI-ICF, pre-substance administration and pre-tDCS.

| **SICI-ICF, pre-substance** | | | | |
| --- | --- | --- | --- | --- |
| **Factor** | **df** | **F value** | **p-value** | **η^2^_p_** |
| Condition | 7 | 0.530 | 0.810 | 0.032 |
| ISI | 1.932^#^ | 208.147 | **<0.001*** | 0.929 |
| Condition × ISI | 7.956^#^ | 0.566 | 0.803 | 0.034 |
| **SICI-ICF, pre-tDCS** |  | | | |
| Condition | 4.783^#^ | 2.324 | 0.053 | 0.120 |
| ISI | 1.746^#^ | 179.157 | **<0.001*** | 0.913 |
| Condition × ISI | 9.829^#^ | 0.876 | 0.555 | 0.049 |

df = degrees of freedom, η^2^p = partial eta squared, ^#^ Greenhouse−Geisser correction according to violation of sphericity; **p* < 0.05

**Table S7.** Results of the two-way ANOVAs for I-wave facilitation, pre-substance administration and pre-tDCS.

| **I-wave facilitation, pre-substance** | | | | |
| --- | --- | --- | --- | --- |
| **Factor** | **df** | **F value** | **p-value** | **η^2^_p_** |
| Condition | 3.988^#^ | 1.037 | 0.395 | 0.057 |
| ISI | 2.045^#^ | 39.518 | **<0.001*** | 0.699 |
| Condition × ISI | 8.356^#^ | 0.693 | 0.946 | 0.039 |
| **I-wave facilitation, pre-tDCS** |  | | | |
| Condition | 7 | 0.904 | 0.506 | 0.050 |
| ISI | 1.783^#^ | 41.306 | **<0.001*** | 0.708 |
| Condition × ISI | 7.835^#^ | 0.655 | 0.968 | 0.37 |

df = degrees of freedom, η^2^p = partial eta squared, ^#^ Greenhouse−Geisser correction according to violation of sphericity; **p* < 0.05

**Table S8.** Results of the repeated measures ANOVA for Single pulse MEP amplitude before and after substance administration (baseline 1 vs baseline 2/3)

| **Factor** | **df** | **F value** | **p-value** | **η^2^_p_** |
| --- | --- | --- | --- | --- |
| **Substance** | 3 | 0.615 | 0.609 | 0.037 |
| **Polarity** | 1 | 0.014 | 0.906 | 0.001 |
| **Time** | 1 | 0.182 | 0.676 | 0.011 |
| **Substance × Polarity** | 3 | 0.367 | 0.777 | 0.022 |
| **Substance × Time** | 3 | 0.124 | 0.946 | 0.007 |
| **Polarity × Time** | 1 | 0.247 | 0.626 | 0.015 |
| **Substance × Polarity × Time** | 3 | 0.727 | 0.541 | 0.043 |

df = degrees of freedom, η^2^p = partial eta squared, **p* < 0.05

**Table S9.** Results of the repeated measures ANOVA for SI1 mV before and after substance administration (baseline 1 vs baseline 2/3)

| **Factor** | **df** | **F value** | **p-value** | **η^2^_p_** |
| --- | --- | --- | --- | --- |
| **Substance** | 1.634^#^ | 1.572 | 0.226 | 0.084 |
| **Polarity** | 1 | 1.795 | 0.197 | 0.095 |
| **Time** | 1 | 3.965 | 0.062 | 0.189 |
| **Substance × Polarity** | 1.827^#^ | 0.495 | 0.597 | 0.028 |
| **Substance × Time** | 3 | 0.150 | 0.928 | 0.008 |
| **Polarity × Time** | 1 | 0.791 | 0.386 | 0.044 |
| **Substance × Polarity × Time** | 3 | 2.667 | 0.057 | 0.135 |

df = degrees of freedom, η^2^p = partial eta squared, ^#^ Greenhouse−Geisser correction according to violation of sphericity; **p* < 0.05

**Table S10.** Results of the repeated measures ANOVA for the I-O curve before and after substance administration (baseline 1 vs baseline 2)

| **Factor** | **df** | **F value** | **p-value** | **η^2^_p_** |
| --- | --- | --- | --- | --- |
| **Substance** | 3 | 0.692 | 0.561 | 0.039 |
| **Polarity** | 1 | 0.700 | 0.414 | 0.039 |
| **Time** | 1 | 1.169 | 0.294 | 0.064 |
| **Intensity** | 1.433^#^ | 521.047 | **<0.001*** | 0.968 |
| **Substance × Polarity** | 3 | 0.398 | 0.754 | 0.022 |
| **Substance × Time** | 3 | 0.188 | 0.903 | 0.010 |
| **Polarity × Time** | 1 | 0.023 | 0.880 | 0.001 |
| **Substance × Polarity × Time** | 2.222^#^ | 0.280 | 0.779 | 0.016 |
| **Substance × Intensity** | 3.128^#^ | 0.458 | 0.720 | 0.026 |
| **Polarity × Intensity** | 1.299^#^ | 0.359 | 0.610 | 0.020 |
| **Substance × Polarity × Intensity** | 3.411^#^ | 0.541 | 0.678 | 0.030 |
| **Time × Intensity** | 1.849^#^ | 0.424 | 0.642 | 0.024 |
| **Substance × Time× Intensity** | 4.564^#^ | 2.278 | 0.060 | 0.118 |
| **Polarity × Time× Intensity** | 1.519^#^ | 1.068 | 0.340 | 0.059 |
| **Substance × Polarity × Time × Intensity** | 4.884^#^ | 0.409 | 0.837 | 0.023 |

df = degrees of freedom, η^2^p = partial eta squared, ^#^ Greenhouse−Geisser correction according to violation of sphericity; **p* < 0.05

**Table S11.** Results of the repeated measures ANOVA for SICI-ICF before and after substance administration (baseline 1 vs baseline 2)

| **Factor** | **df** | **F value** | **p-value** | **η^2^_p_** |
| --- | --- | --- | --- | --- |
| **Substance** | 2.010^#^ | 2.362 | 0.109 | 0.122 |
| **Polarity** | 1 | 0.777 | 0.390 | 0.043 |
| **Time** | 1 | 3.744 | 0.069 | 0.180 |
| **ISI** | 1.439^#^ | 193.164 | **<0.001*** | 0.919 |
| **Substance × Polarity** | 3 | 0.062 | 0.979 | 0.003 |
| **Substance× Time** | 2.275^#^ | 5.104 | **0.008*** | 0.230 |
| **Polarity × Time** | 1 | 0.011 | 0.632 | 0.013 |
| **Substance× Polarity × Time** | 3 | 0.384 | 0.764 | 0.022 |
| **Substance × ISI** | 6.629^#^ | 0.964 | 0.457 | 0.053 |
| **Polarity × ISI** | 2.799^#^ | 1.112 | 0.351 | 0.061 |
| **Substance× Polarity× ISI** | 5.029^#^ | 0.335 | 0.891 | 0.019 |
| **Time× ISI** | 5 | 2.303 | 0.051 | 0.119 |
| **Substance × Time× ISI** | 7.243^#^ | 1.430 | 0.196 | 0.077 |
| **Polarity × Time × ISI** | 5 | 0.764 | 0.578 | 0.043 |
| **Substance × Polarity × Time × ISI** | 6.447^#^ | 0.496 | 0.821 | 0.028 |

df = degrees of freedom, η^2^p = partial eta squared, ^#^Greenhouse−Geisser correction according to violation of sphericity; **p* < 0.05

**Table S12.** Results of the repeated measures ANOVA for I-wave facilitation before and after substance administration (baseline 1 vs baseline 2)

| **Factor** | **df** | | **F value** | **p-value** | **η^2^_p_** |
| --- | --- | --- | --- | --- | --- |
| **Substance** | | 3 | 2.345 | 0.083 | 0.121 |
| **Polarity** | | 1 | 0.836 | 0.373 | 0.046 |
| **Time** | | 1 | 0.042 | 0.838 | 0.002 |
| **ISI** | | 1.623^#^ | 46.106 | **<0.001*** | 0.730 |
| **Substance × Polarity** | | 3 | 0.539 | 0.657 | 0.030 |
| **Substance× Time** | | 1.388^#^ | 0.108 | 0.824 | 0.006 |
| **Polarity × Time** | | 1 | 0.001 | 0.967 | 0.000 |
| **Substance× Polarity × Time** | | 3 | 0.386 | 0.763 | 0.022 |
| **Substance × ISI** | | 5.256^#^ | 0.943 | 0.460 | 0.052 |
| **Polarity × ISI** | | 2.254^#^ | 0.398 | 0.698 | 0.022 |
| **Substance× Polarity× ISI** | | 4.198^#^ | 0.446 | 0.783 | 0.025 |
| **Time× ISI** | | 7 | 0.917 | 0.495 | 0.051 |
| **Substance × Time× ISI** | | 8.016^#^ | 0.621 | 0.758 | 0.035 |
| **Polarity × Time × ISI** | | 7 | 0.500 | 0.832 | 0.028 |
| **Substance × Polarity × Time × ISI** | | 8.561^#^ | 1.055 | 0.398 | 0.058 |

df = degrees of freedom, η^2^p = partial eta squared, ^#^ Greenhouse−Geisser correction according to violation of sphericity; **p* < 0.05

**Table S13.** Results of the repeated measures ANOVA conducted for I-wave facilitation before

and after tDCS

| **Factor** | **df** | **F value** | **p-value** | **η^2^_p_** |
| --- | --- | --- | --- | --- |
| **Substance** | 3 | 0.830 | 0.483 | 0.046 |
| **Polarity** | 1 | 2.528 | 0.130 | 0.129 |
| **Time** | 2 | 21.103 | **<0.001*** | 0.553 |
| **ISI** | 1.604^#^ | 66.680 | **<0.001*** | 0.796 |
| **Substance × Polarity** | 3 | 0.750 | 0.527 | 0.042 |
| **Substance × Time** | 6 | 1.089 | 0.374 | 0.060 |
| **Polarity × Time** | 2 | 4.342 | **0.020*** | 0.203 |
| **Substance × Polarity × Time** | 6 | 1.117 | 0.357 | 0.061 |
| **Substance × ISI** | 5.920^#^ | 0.529 | 0.958 | 0.030 |
| **Polarity × ISI** | 3.033^#^ | 0.752 | 0.527 | 0.042 |
| **Substance × Polarity × ISI** | 4.244^#^ | 0.415 | 0.807 | 0.023 |
| **Time × ISI** | 14 | 2.599 | **0.002*** | 0.132 |
| **Substance × Time× ISI** | 10.365^#^ | 1.334 | 0.213 | 0.072 |
| **Polarity × Time× ISI** | 14 | 1.984 | **0.019*** | 0.104 |
| **Substance × Polarity × Time × ISI** | 10.546^#^ | 1.537 | 0.125 | 0.082 |

df = degrees of freedom, η^2^p = partial eta squared, ^#^Greenhouse−Geisser correction according to violation of sphericity; **p* < 0.05


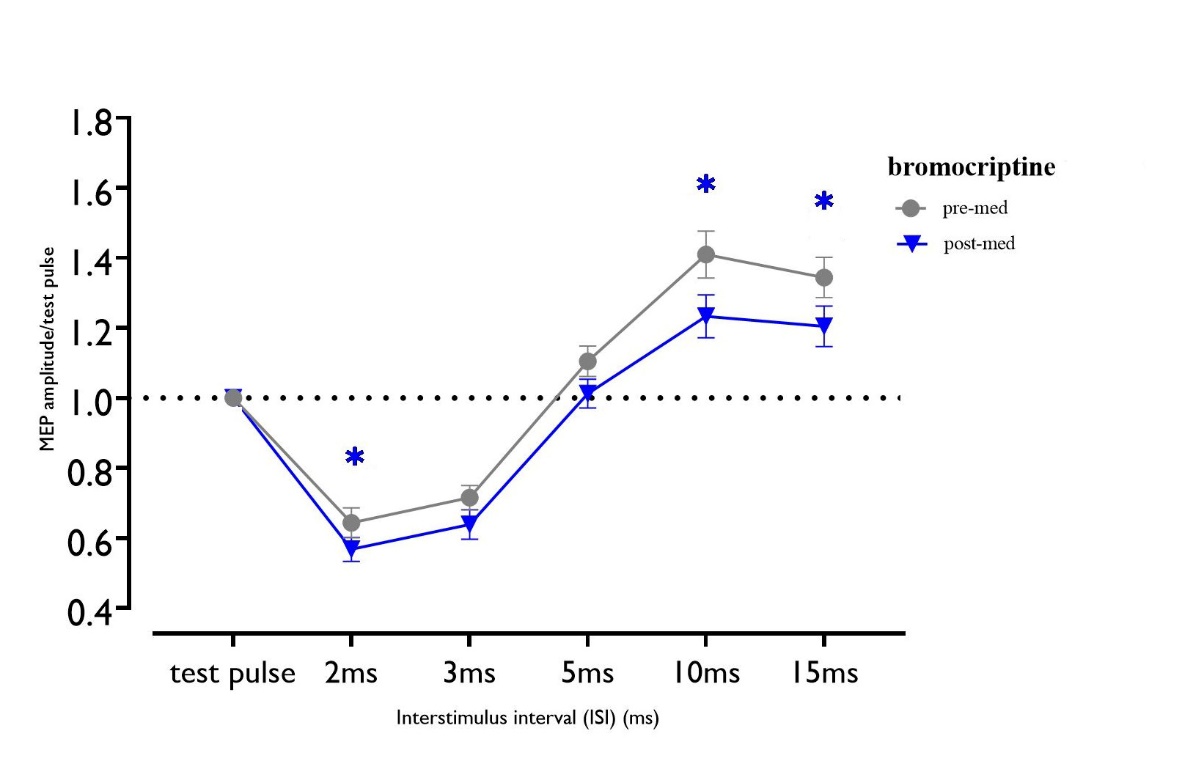


**Figure S1: SICI-ICF before and after substance use (BL1 VS BL2) in the bromocriptine condition.**

Bromocriptine increased SICI at ISI 2ms and decreased ICF at ISIs 10 and 15 ms post-substance as compared to the pre-substance condition. Blue asterisks indicate significant differences between the pre- and post-bromocriptine intake conditions (*) based on Fisher's LSD post-hoc comparisons, *p* <.05. Error bars represent standard error of the mean.
